# Accurate exclusion of kidney regions affected by susceptibility artifact in blood oxygenation level-dependent (BOLD) images using deep-learning-based segmentation

**DOI:** 10.1038/s41598-023-46760-2

**Published:** 2023-11-06

**Authors:** Chang Ni, Xin Mu, Mingyan Wu, Yanbin Li, Yuyao Zhang, Haikun Qi, Jeff L. Zhang

**Affiliations:** 1 School of Biomedical Engineering, ShanghaiTech University, Room 416, BME Building, 393 Middle Huaxia Road, Pudong, Shanghai, China; 2 Central Research Institute, United Imaging Healthcare Group, Shanghai, China; 3 School of Information Science and Technology, ShanghaiTech University, Shanghai, China

**Keywords:** Kidney, Magnetic resonance imaging, Biomedical engineering

## Abstract

Susceptibility artifact (SA) is common in renal blood oxygenation level-dependent (BOLD) images, and including the SA-affected region could induce much error in renal oxygenation quantification. In this paper, we propose to exclude kidney regions affected by SA in gradient echo images with different echo times (TE), based on a deep-learning segmentation approach. For kidney segmentation, a ResUNet was trained with 4000 CT images and then tuned with 60 BOLD images. Verified by a Monte Carlo simulation, the presence of SA leads to a bilinear pattern for the segmented area of kidney as function of TE, and the segmented kidney in the image of turning point’s TE would exclude SA-affected regions. To evaluate the accuracy of excluding SA-affected regions, we compared the SA-free segmentations by the proposed method against manual segmentation by an experienced user for BOLD images of 35 subjects, and found DICE of 93.9% ± 3.4%. For 10 kidneys with severe SA, the DICE was 94.5% ± 1.7%, for 14 with moderate SA, 92.8% ± 4.7%, and for 46 with mild or no SA, 94.3% ± 3.8%. For the three sub-groups of kidneys, correction of SA led to a decrease of R_2_* of 8.5 ± 2.8, 4.7 ± 1.8, and 1.6 ± 0.9 s^−1^, respectively. In conclusion, the proposed method is capable of segmenting kidneys in BOLD images and at the same time excluding SA-affected region in a fully automatic way, therefore can potentially improve both speed and accuracy of the quantification procedure of renal BOLD data.

## Introduction

As arguably the only method that is capable of non-invasively measuring tissue oxygenation, blood oxygenation level-dependent (BOLD) MRI has been widely investigated for assessing potential renal hypoxia in various kidney diseases^1–4^. For example, BOLD-measured R_2_* in renal medulla significantly increased in patients with diabetic nephropathy^5^ and with renal artery stenosis^6,7^. To avoid confounding impacts from varying factors in different patient populations, efforts were also devoted to quantifying tissue oxygenation from renal BOLD signals^8,9^. After more than 2 decades of developments, a worldwide group of renal BOLD investigators recently recommended a consensus protocol for renal BOLD’s image acquisition and processing, with the hope of accelerating the method’s clinical adoption^10^.

A significant problem with renal BOLD is susceptibility artifact (SA), which may severely degrade image quality for a large region of kidneys. In the magnetic field, field inhomogeneity forms around the boundary between two adjacent media with different magnetic susceptibility, e.g. tissue versus air, and such field inhomogeneity may be of quite high magnitude and propagate far from the boundary^9,11^. Due to the high field inhomogeneity, the regions affected with SA typically have very high R_2_* and thus very fast signal decay with echo time (TE), or local dimming effect. For the kidneys, a major source of SA is the presence of bowel gas, which predominantly affects the lower-left lobe of the left kidney^12–14^. There is no solution for fully eliminating susceptibility artifacts in BOLD acquisitions. In post-processing, one typically first visually check the BOLD images of different TEs, and the renal regions that show local dimming are identified as SA-affected and would be excluded from renal cortex or medulla regions of interest (ROI) in further analysis. Apparently, such an empirical method for identifying SA may be erroneous and produce different results by different users.

**Figure 1:**
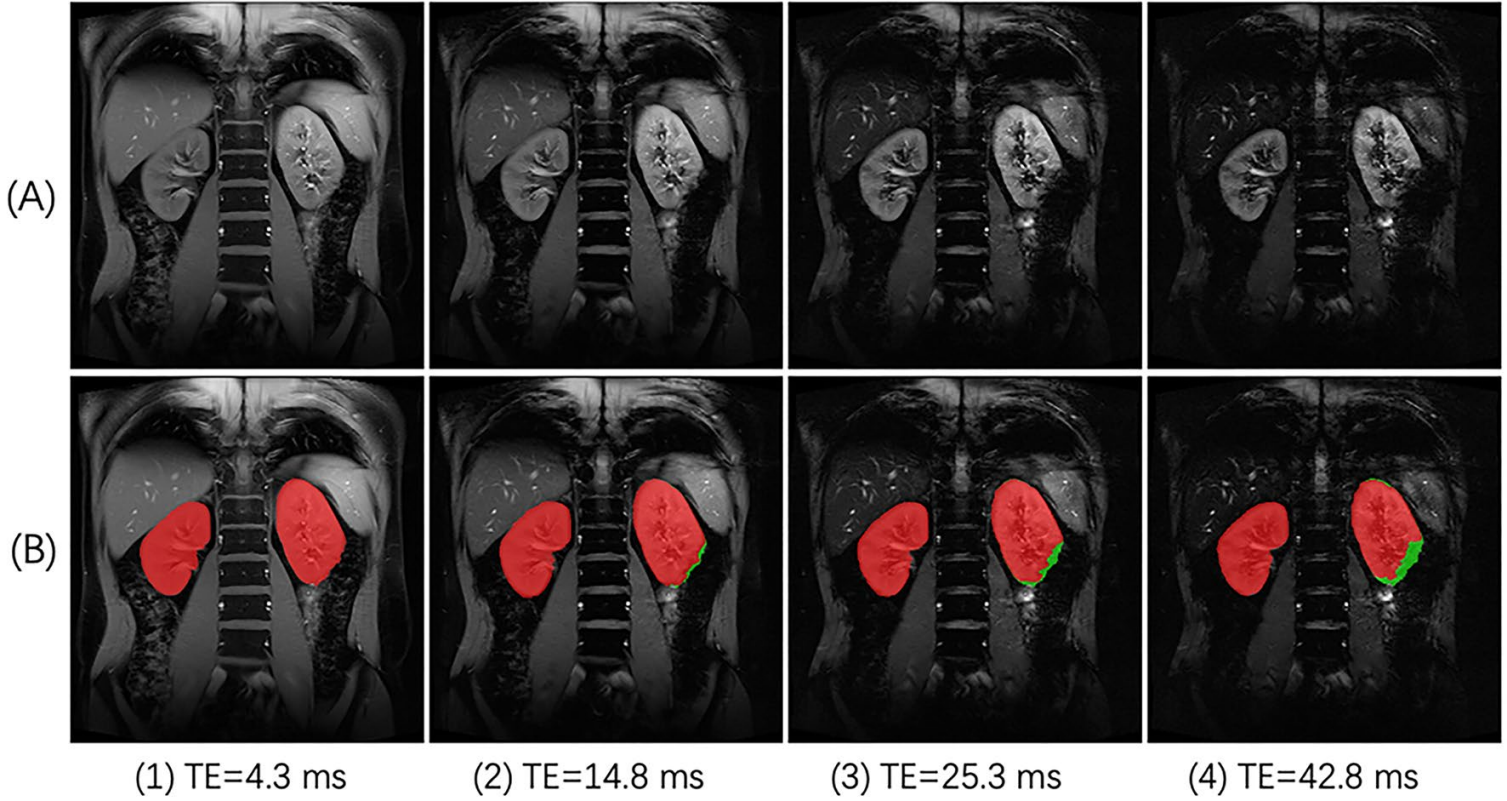
An example of renal BOLD images affected by susceptibility artifact (SA). (**A**) BOLD images acquired with TE of different values. The dimming region expanded from the lower lobe of the left kidney. (**B**) ROIs of the kidneys for further analysis were manually delineated as red masks, and SA-affected regions were also determined and shown as green masks.

Deep learning for image segmentation evolves fast in the past few years, and may help identify SA-affected regions of the kidneys. Different from most traditional methods that rely on individual image features such as image intensities and shapes, deep learning methods segment images based on high-order features learned from large sets of images of similar type, i.e., training data. As a result, deep learning methods typically perform quite robustly in segmenting images with less-predicted intensity range or ROI shapes^15–17^, one such challenging example being our case of renal BOLD images acquired at different TE. Signal decay with increased TE creates multiple images with a wide range of signal intensity. With different R_2_* values in renal cortex, medulla, and SA-affected region, signal intensity in these regions decay with different rates, so that a kidney may display shapes of quite a difference in images of different TE (Fig. 1).

**Figure 2:**
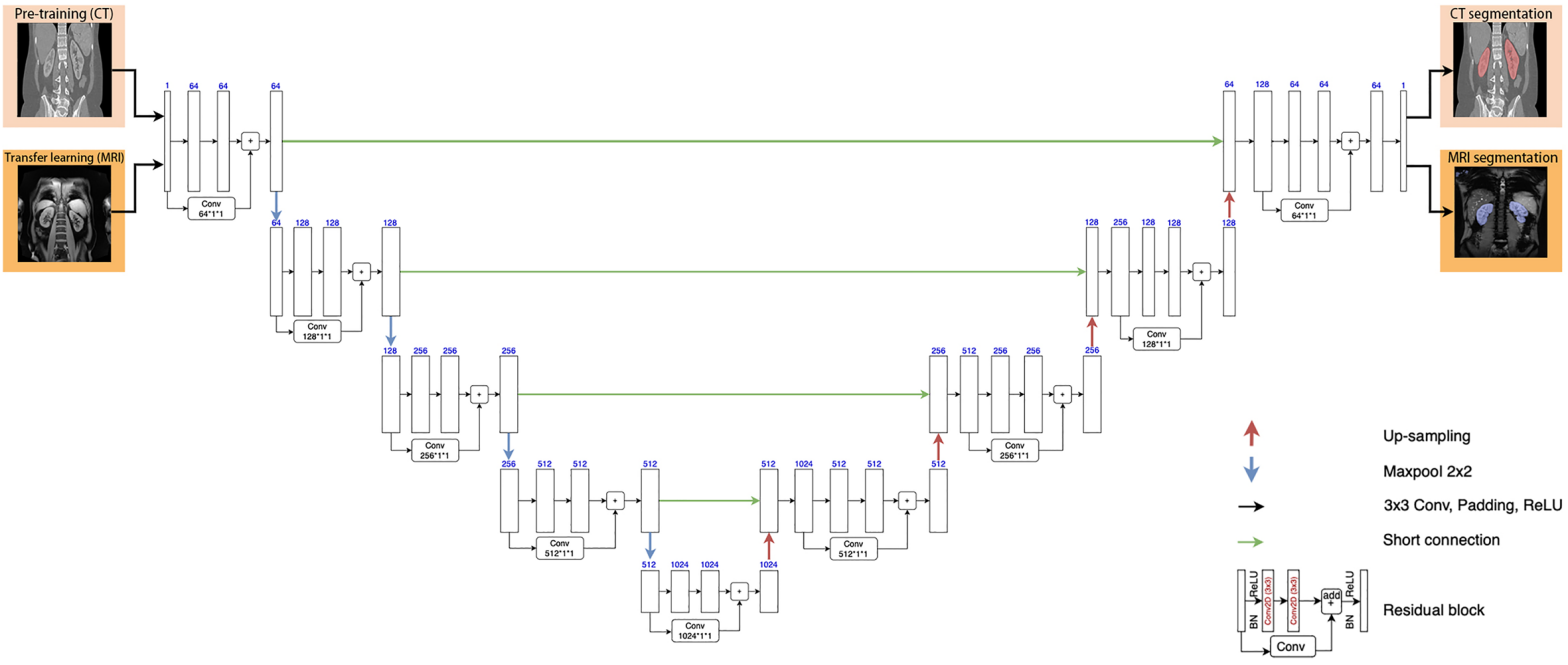
The ResUNet model architecture consisted of an encoder pathway (the left side) and a decoder pathway (the right side), each containing 4 blocks of down-sampling or up-sampling coupled with residual learning. Pre-training was done with CT data (top row of images), followed by fine-tuning all the layers with a small set of MRI images (second row of images).

In this study, we trained a convolutional neural network (CNN) (Fig. 2) for segmenting kidneys in multi-TE BOLD images. Based on the segmented volumes, we proposed an automatic method for accurately identifying SA-affected regions in the segmented kidneys.

## Results

### Accuracy of kidney segmentation

**Figure 3:**
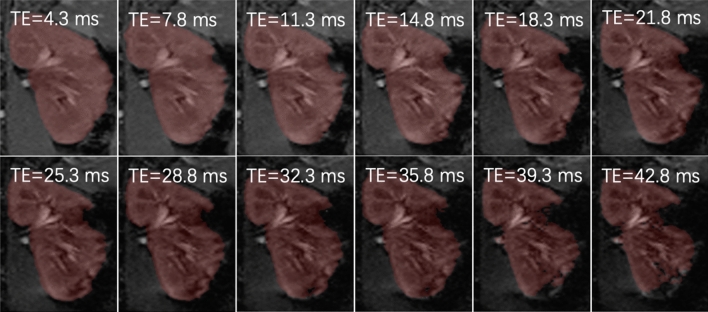
An example of segmenting multi-gradient-echo images of kidney using the proposed method of ResUNet and transfer learning. The segmented mask in red captured the kidney region as its signal decayed with TE and excluded the expanding SA-affected region.

The deep-learning-based segmentation performed comparably with manual segmentation in segmenting 420 kidney BOLD images with different TE values and different degrees of SA, with DICE of 93.9% ± 3.4%. One example of segmentation is shown in Fig. 3, demonstrating the method’s capability in segmenting images with different contrast between kidney and background and with SA.

### Simulation to verify the biphasic decrease of segmented kidney volume with TE

**Figure 4:**
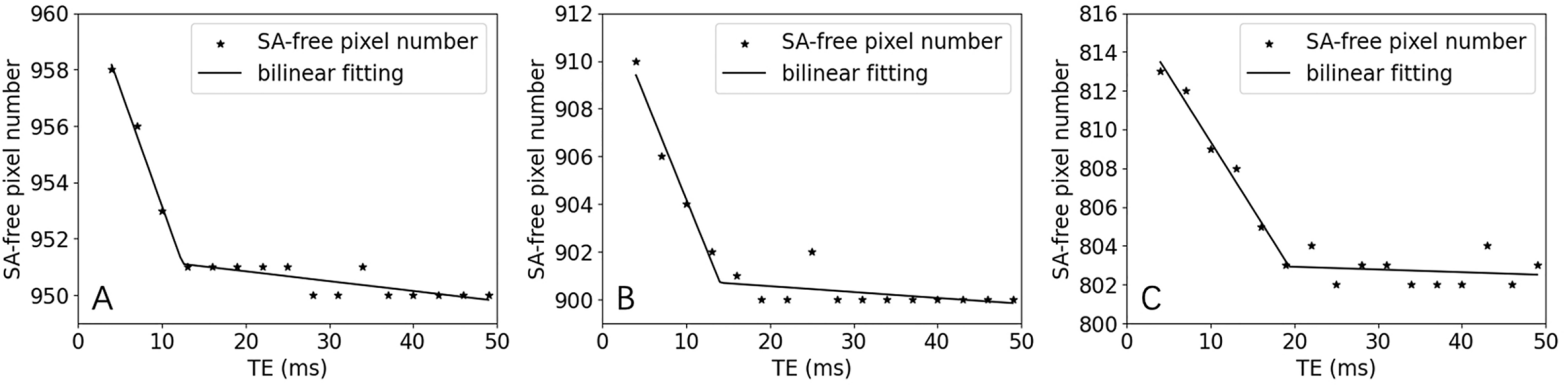
Representative examples of simulated classified number of SA-free pixels versus TE curves for different fractions of SA-affected pixels, and their bilinear fitting. (**A**) SA-affected fraction of 5%, or 950 SA-free pixels; (**B**) SA-affected fraction of 10%, or 900 SA-free pixels; (**C**) SA-affected fraction of 10%, or 800 SA-free pixels.

A biphasic pattern was observed in segmented kidney volume versus TE curves of human-subject data, which motivated us to perform a simulation for verification. Figure 4 shows three example curves of simulated kidney segmentation volume versus TE that correspond to SA-affected fractions of 5%, 10%, and 20%, respectively. The curves show clear biphasic pattern, with an initial quick downslope followed by a much slower downslope. Across the 100 simulation trials for each volume fraction, relative RMS by the bilinear fitting was averaged to be 0.11% ± 0.04%, 0.16% ± 0.07%, and 0.25% ± 0.09%, respectively, and the estimated number of SA-free pixels was 951.0 ± 1.0, 902.2 ± 1.5, and 804.1 ± 1.9, very close to their respective true value of 950, 900 and 800 voxels.

### Application of the bilinear fitting approach to data of human subjects

**Figure 5:**
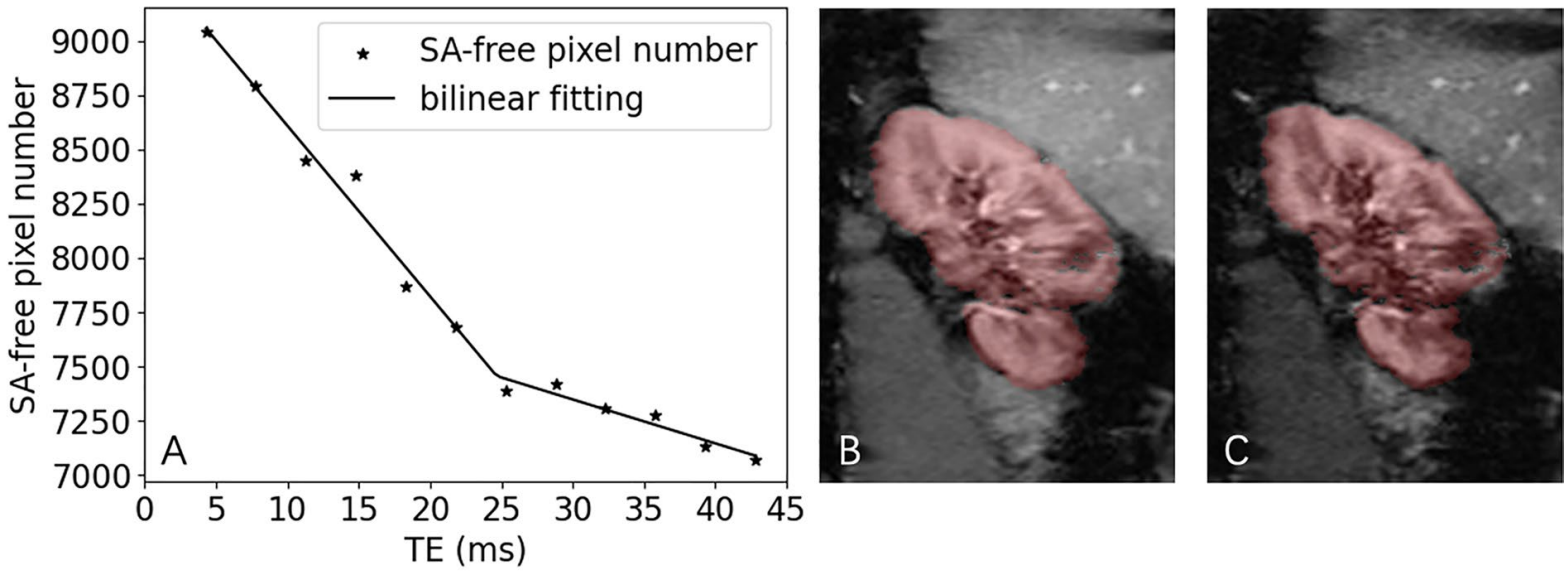
A representative example of application of the proposed method to a human kidney BOLD data. (**A**) The segmented kidney area versus TE curve with bilinear fitting; area affected by SA was 17.6%, and the relative RMS of the bilinear fitting was 0.42%. (**B**) The image with TE of 25.3 ms (estimated turning point), with overlayed mask (in red). (**C**) The image with TE of 43.8 ms (the longest TE for the acquired set of images), with overlayed mask (in red). In the lower-right lobe, signal decay made the kidney tissue non-differentiable from the background.

Figure 5 shows a representative example of segmented kidney area versus TE curve for a human kidney with an SA-affected fraction of 17.6%. The relative RMS of bilinear fitting for this case was 0.42%. The DICE between the SA-excluded ROI by this method and by the manual method was 95.4%.

For the 35 BOLD data, all the 70 kidneys were included in the analysis, among which 46 had no or mild SA (affected fraction 2.1% ± 1.4%), 14 moderate SA (affected fraction 6.6% ± 1.2%), and the other 10 severe SA (affected fraction 14.3% ± 3.9%). For the three groups, the RMS of bilinear fitting was 18.1 ± 6.4, 27.2 ± 8.6 and 51.5 ± 13.1 voxels, respectively, and the corresponding relative RMS was 0.19% ± 0.07%, 0.31% ± 0.08%, and 0.58% ± 0.17%. When comparing the SA-excluded ROIs by the proposed method and by the manual method, the DICE coefficient was 94.3% ± 3.8%, 92.8% ± 4.7%, and 94.5% ± 1.7%, for the groups of kidneys with mild, moderate and severe SA, respectively. For the three groups, the SA-excluded ROIs by the proposed method were − 0.8% ± 4.7%, 1.1% ± 5.2%, and − 3.5% ± 4.4%, larger than those by the manual segmentation, respectively. Exclusion of the SA-affected regions from kidney ROI resulted in significantly different or more accurate R_2_* values (*P* < 0.001). Between ROIs corrected for SA and those not corrected, the ROI-averaged R_2_* values differed by 1.6 ± 0.9, 4.7 ± 1.8 and 8.5 ± 2.8 s^−1^, for the three groups, which account for 5.1% ± 2.7%, 14.7% ± 5.3% and 23.4% ± 7.6% of the averaged kidney R_2_* value, respectively (Table 1).

**Table 1:** Application of the proposed method to correct SA for 70 kidneys: 46 with no or mild SA, 14 with moderate SA, and 10 with severe SA.

	Kidneys with mild SA	Kidneys with moderate SA	Kidney with severe SA	All the kidneys
RMS (voxels)	18.1 ± 6.4	27.2 ± 8.6	51.5 ± 13.1	29.2 ± 16.4
Relative RMS (%)	0.19% ± 0.07%	0.31% ± 0.08%	0.58% ± 0.17%	0.32% ± 0.19%
SA-excluded ROI DICE	94.3% ± 3.8%	92.8% ± 4.7%	94.5% ± 1.7%	93.9% ± 3.4%
R_2_* decrease after SA correction (s^−1^)	1.6 ± 0.9	4.7 ± 1.8	8.5 ± 2.8	4.3 ± 3.4
R_2_* decrease after SA correction (%)	5.1% ± 2.7%	14.7% ± 5.3%	23.4% ± 7.6%	12.6% ± 9.1%

The performance of the method was evaluated based on the goodness of fit (RMS and relative RMS) of bilinear fitting, DICE of ROI as compared to that by the manual method, and the averaged R_2_* values over kidney ROI.

## Discussion

Susceptibility artifact occurs commonly in imaging kidney with BOLD, and failure to exclude it from kidney’s ROI may lead to a significant overestimation of averaged R_2_* values. In this study, we utilized a robust deep-learning segmentation technique to automatically and accurately exclude SA from multi-gradient-echo images. The method performed comparably with the time-consuming manual method, with outcome similarity of ~ 94% across different degrees of SA. Some differences between the ROIs by the two methods were noticed, particularly for the severe-SA kidneys, with the proposed method’s ROIs about 3.5% smaller than those by manual segmentation. Such discrepancy was presumably attributed to the capability of the proposed method in excluding more regions with minor SA. With the SA-affected region excluded from kidney ROI, a fraction of 15–25% can be corrected from the overestimated ROI-averaged R_2_* values. For implementation, the method involves only running segmentation with a trained deep learning model and followed by a linear fitting, so can be easily incorporated into the post-processing workflow of kidney BOLD images.

The core of the proposed method is reliable segmentation of kidney region excluding SA-affected region that was achieved by the deep-learning-based approach. Such segmentation is quite challenging, as gradient echo signals from kidney tissue decay with TE, with quite different rates. For example, at 3 T, renal medulla’s R_2_* can be as high as 30–40 s^−1^, while that in renal cortex is only around 15 s^−1^. Images acquired at different TE values would display different patterns, from a homogeneous kidney shape at low TE to renal cortex only at relatively high TE, and almost no contrast between kidney and background at very high TE. Another layer of complexity is brought by SA, R_2_* of which is estimated to be higher than 50 s^−1^, meaning that the SA-affected regions dim and merge with the dark background since low TE values. The ideal segmentation method should handle both the varying contrast between the kidney and background and the evolvingly complex shapes of the kidney region across different TE values. None of the traditional segmentation methods, either region-based or shape-based, could achieve such segmentation reliably. Our ResUNet model was first trained with a large set of post-contrast arterial-phase CT images. As the images had a variety of degrees of cortico-medullary contrast (depending on kidney’s function) and the provided labels for model training covered both cortex and medulla, the trained model had the capability of segmenting kidney in BOLD images of different TE values. Further tuning of the model with a small set of BOLD images presumably calibrated for any difference in image intensity between CT and BOLD images. While we did see a biphase pattern in curves of segmented kidney volume versus TE for patient data, the second phase at long TE values was mostly not flat as expected but displayed a slow decrease (Fig. 5). Such decrease was caused by the shrinking of segmented masks for the images of long TEs; the contrast between kidney and background diminishes with the increase of TE, particularly in the outer region of a kidney with partial volume artifact. This is why we propose to find the curve’s turning point, where the SA-affected region is maximally excluded and the non-affected region is maximally preserved.

Our biphasic hypothesis for the segmented volume versus TE curve was based on empirical observations with multi-gradient echo images. At kidney region without SA, signal decay with TE is due to intravoxel deoxyhemoglobin, with a relaxation rate R_2_* of around 15–30 s^−1^. To precisely measure R_2_* of such range, images are acquired at multiple TE values, with maximal TE typically set at 50–80 ms. Take a voxel with R_2_* of 20 s^−1^ as an example. From TE of 0–60 ms, the voxel’s signal would decay from 100 to 30% of its initial value. SA is caused by magnetic field heterogeneity propagated from outside of kidneys, e.g. bowel gas, and leads to R_2_* on the order of 100 s^−1^ or higher. For the examples shown in Figs. 1 and 3, the SA-affected regions’ signal intensity decreased to a background level as early as TE of 10–15 ms. As expected, in images of TE 0–60 ms, the SA-affected region would dim quickly to the intensity of the background, causing the initial rapid decrease in the segmented volume versus TE curve. With a simulation, we validated the bilinear pattern for different severity degrees of SA.

An alternative way of excluding SA is to generate R_2_* map first, and then exclude the regions with extraordinarily high R_2_* values. While this approach can also be implemented automatically by directly segmenting the R_2_* map, it has two potential problems. First, depending on the method used for fitting BOLD signals to estimate voxelwise R_2_*, background surrounding a kidney can be quite complex, with either very high or low R_2_* level, making reliable segmentation impossible. In the original BOLD images that our method applies to, presence of SA can decay background signals further, making the segment of kidney and background easier. Second, for cases with moderate or severe SA, the artifact could propagate to more inner regions such as the inner medulla that has a high R_2_* level; it can be quite challenging for either the human operator or any algorithm to determine a confident boundary for the SA propagation. This is nicely solved in the proposed method, in which area of the SA-affected region is tracked and linearly extrapolated from BOLD image of the very first TE.

This study has multiple limitations. First, the segmentation model was trained with coronal images, so it won’t work well for axial or sagittal images; however, the methodology would be the same, and the same set of CT images can be re-formulated to either axial or sagittal view if needed. Second, the proposed method identifies and excludes the regions affected by SA, but not corrects the artifact. As a majority of kidney-BOLD applications are CKD-related, potential alteration in tissue hypoxia is homogeneous in those diseases. Third, the proposed method involves a bilinear fitting, for which an adequate number of TE values are necessary. In other words, given images of only a few TE values, the method won’t work well. In this study, we did not perform analysis on the minimal number of TE values for an acceptable outcome of SA exclusion.

## Conclusion

Based on a robust deep-learning model for image segmentation, the proposed method automatically and accurately segments kidney and excludes possible SA-affected region in renal BOLD images. Compared to the currently used manual method, the proposed method would dramatically improve the efficiency and reliability of renal BOLD analysis. Future studies could further validate the proposed method on larger datasets and explore its applicability in clinical settings.

## Materials and methods

To automatically exclude SA-affected region from kidney ROI, we propose a method that mimics how a user would manually perform the task. The manual approach is performed in the following manner: given a set of BOLD or gradient echo images with different TE, one visually checks every image, localizing each kidney; a part of kidney is suspected of SA, if the region has intensity much lower than its surrounding kidney region; if the locally dimming region expands as TE increases, then it is SA-affected (Fig. 1) and the expanded region should be excluded from further analysis. This empirical method is based on the fact that SA causes field inhomogeneity of magnitude much higher than deoxyhemoglobin-induced BOLD effect, and therefore leads to much faster signal decay than renal tissue. Expansion of the above-mentioned dark region typically starts from kidney’s outer boundary, as SA is mostly propagated from other organs such as bowel gas.

Following the methodology of the manual method, the proposed automatic method first uses CNN-based segmentation to segment kidney from its background. The segmentation should be robust enough for segmenting kidneys with different contrasts (as in images of different TE) against its background, and for kidneys with irregular contour due to SA. We achieved such segmentation using a ResUNet trained with a large set of kidney CT images and further tuned with a small set of BOLD MRI images, i.e. transfer learning. Due to the expansion of SA-affected area with increased TE, segmented kidney volume^18^ should initially decrease with TE, and then stay stable after some TE value. To verify this biphasic pattern of segmented volume versus TE curve, we performed a simulation study that simulated signal decay of a large set of tissue voxels and classified signal intensity of the voxels without and with the impact of SA as an approximation of segmentation. Fitting the verified biphasic pattern with a bilinear function would enable us to identify the TE value at which expansion of SA-affected area stops, or the maximal SA-affected region to be excluded from kidney ROI. Note that images of very large TE are bad candidates for SA exclusion because with strong signal decay at large TE, kidney region is hard to be accurately segmented from background. For validating the proposed method, we compared kidney ROI delineated by the proposed method against that from manual segmentation for a group of BOLD images with different degrees of SA.

### Kidney segmentation with ResUNet and transfer learning

To segment kidney BOLD images, we chose a CNN model well known for image-segmentation tasks, deep residual UNet or ResUNet (Fig. 2). To fully characterize the relatively complex features of kidney contour, we pre-trained the ResUNet model with a large set of kidney CT images, and then using transfer learning technique, fine-tuned the model with a small set of kidney BOLD images.

The ResUNet model has a similar two-pathway structure as UNet, i.e. an encoder pathway and a decoder pathway (Fig. 2). Each pathway contains 4 blocks of down-sampling or up-sampling coupled with residual learning. The residual block in each down- or up-sampling block is built with two successive 3 × 3 convolutional blocks and one identity mapping. Each convolution block includes a batch normalization layer, a Rectified Linear Unit (ReLU) activation layer, and a convolutional layer. The identity mapping connects the input and output of the residual block. A max-pooling layer is applied to reduce the spatial dimension of the feature maps by half at the down-sampling encoder block and up-sampling is applied in the corresponding decoder block. With the residual blocks, ResUNet solves the degradation problem with UNet, resulting in improved channel inter-dependency and computational efficiency^19,20^.

The ResUNet model was pre-trained with 4000 kidney CT images acquired during the arterial phase after contrast injection. The images along with their kidney segmentation by experienced experts were obtained from the website of the KiTS19 challenge (https://kits19.grand-challenge.org). Images of 200 subjects were acquired axially, with a matrix of 512 × 512 and slice thickness from 1 to 5 mm. We reformatted all the images into coronal view, and from each set of coronal images selected 20 images that covered the kidneys, resulting in 4000 coronal images. Each of the images was trimmed or padded to matrix size of 512 × 512, and intensity was normalized to the range between 0 and 1. Each image was augmented with a random transform method provided by Albumentations^21^. For model training, we used the Adam optimizer to minimize the pixel-wise cross-entropy loss function, batch size of 1, and epochs of 200. In each epoch, randomly-sampled images from the training image set were fed into the network. The learning rate was initially set to 1e−4 and decayed with the ReduceLROnPlateau method. The training was implemented on a PC workstation equipped with NVIDIA GeForce RTX 3070 GPU (8 Gb) and Inter(R) Xeon(R) Gold 5220R CPU (frequency 2.20 GHz, 48 cores), and in an environment with Ubuntu 20.04.3 LTS OS and the CUDA version 11.6.

The above-trained model presumably learned features such as the location and shape of kidneys in the CT images. Such learned features were transferred by further training the model with a small set of kidney BOLD images. The BOLD images were acquired with a 3 T MRI scanner from 5 healthy subjects; each subject’s dataset contained 12 coronal images with TE from 4.3 to 42.8 ms (see more details of the data in the following section), so the total number of BOLD images for model tuning was 60. Of the 5 subjects’ data, 3 were used for model training, and the other 2 for model validation. Reference kidney masks in these images were manually annotated by experienced users using LabelMe^22^. The fine-tuning procedure was performed with the same training specifications as in the pre-training, except for the higher epochs of 1000 needed for the smaller training set.

To test the performance of the segmentation method, we compared it against a manual method in segmenting kidney BOLD images acquired for 35 subjects. All the MRI experiments were approved by the Research Ethics Committee of ShanghaiTech University (ShanghaiTech BME IRB#2021-008), and before the experiments all the subjects signed written informed consent forms. All the data were acquired on clinical 3 T MRI scanners (12 on Tim Trio, Siemens Medical Solutions; 23 on uMR890, United Imaging Health), using gradient echo sequence with a similar set of protocol^23^: 2D single coronal slice, slice thickness 7 mm, field of view 420 × 336 mm, matrix 320 × 272, bandwidth 300 Hz/pixel, 12 TE from 4.3 to 42.8 ms, repetition time 70 ms, flip angle 30°, fat suppression ON. For the overall 420 images, masks for the left and the right kidneys were obtained by the proposed method, and by manual delineation by an experienced user, independently. The two sets of kidney masks were compared by computing the paired DICE, i.e. two times the overlap area of two masks divided by the sum of the two masks’ area. DICE of two perfectly matched masks is 1, and of two fully separate ones is 0.

### Automatic exclusion of SA-affected region with a biphasic fitting method

While SA may cause field inhomogeneity for a large region of kidneys, the induced dimming in gradient echo images shows up fully when TE is long enough. Our preliminary study showed that with SA, segmented kidney volume versus TE curve would show a pattern of biphasic decay, with the initial fast decay presumably due to SA. To verify this biphasic pattern, we conducted a simulation that simplified the segmentation of multi-gradient echo images with SA into a classification of tissue voxels with signal decay of two different rates, i.e. with or without SA. After verification of the biphasic pattern, we introduced a biphasic fitting method that automatically identifies the TE value of the image in which the SA-affected region is fully excluded from the segmented kidney mask.

In a simulation, we simplified the segmentation of SA-affected kidneys in multi-gradient echo images into a classification problem. A set of 1000 pixels were simulated, a fraction of which was assumed to be affected by SA. The SA-affected pixels were assigned with R_2_* values randomly sampled from a Gaussian distribution. Mean and standard deviation of the distribution were selected so that 95% of the sampled values were in the range of 50–200 s^−1^ (typically observed range for SA at B_0_ of 3 T). For the pixels free of SA, R_2_* values were sampled from the typical range of renal cortex at 3 T, i.e. 16–18 s^−1^. For every pixel, gradient-echo signal intensities were generated for 16 TE values from 0 to 50 ms with an interval of 3 ms, using the exponential decay formula S = S_0_·exp(− TE·R_2_*). The initial signal intensity S_0_ was assumed to be 100. For every simulated signal, random Rician noise of 5% was added. K-means classification^24^ was applied to classify every set of 1000 pixels’ simulated signals at each TE value, to obtain a curve of classified number of SA-free pixels versus TE. We repeated the above process 100 times, for each of the three chosen fractions of SA-affected pixels: 5%, 10%, and 20%, representing mild, moderate, and severe degree of SA, respectively. We fitted each curve of SA-free-pixel number versus TE using a bilinear function^25^, implemented with a segmented regression approach^26^. After each fitting, we recorded the x- and y-coordinates of the turning point of the bilinear fit. The x-coordinate of the turning point corresponds to the minimal TE value at which SA fully shows up in an image, and the y-coordinate would be the estimated number of SA-free pixels. We compared the estimated number of SA-free pixels against the actual number of SA-free pixels, i.e. 950, 900, or 800, and the error was averaged across the 100 simulation trials for each of the three SA-affected fractions. We also computed root mean square (RMS) error between the linear fit and the classified numbers of SA-free pixels for evaluating goodness of fit, and then computed the mean and the standard deviation of the 100 RMS errors for each of the three SA-affected fractions. Low fitting residue would indicate the validity of the biphasic pattern of SA-free volume versus TE, and also the feasibility of fitting it bilinearly.

For the same 70 kidneys for testing the segmentation method, we applied the above bilinear fitting approach to determine the TE value of the turning point in their segmented volume versus TE curves. As TE values for the BOLD data were discrete with a gap of 3.5 ms, we selected the image with TE value closest to the fitted TE value, and used the segmented ROI in this image as the kidney ROI with excluded SA region. This SA-excluded ROI was compared against the ROI determined by an experienced user, by computing the DICE of the two ROIs. The evaluation was performed separately for kidneys with mild (< 5%), moderate (5–10%), and severe SA (> 10%). In addition, we compared R_2_* values of the entire kidney ROI (without excluding the SA-affected region) and of the kidney ROI with SA correction, using a paired *t*-test. We hypothesized that with the SA-affected region excluded, the ROI-averaged R_2_* value would significantly decrease. All of the above processing programs were implemented using MATLAB (MathWorks, Natick, MA).

## Data Availability

The datasets used and analyzed during the current study are available from the corresponding author on reasonable request.
